# Cold acclimation and health: effect on brown fat, energetics, and insulin sensitivity

**DOI:** 10.1186/2046-7648-4-S1-A45

**Published:** 2015-09-14

**Authors:** Wouter D van Marken Lichtenbelt, Mark JW Hanssen, Joris Hoeks, Anouk AJJ van der Lans, Boudewijn Brans, Felix M Mottaghy, Patrick Schrauwen

**Affiliations:** 1 Department of Human Biology, NUTRIM School for Nutrition, Toxicology and Metabolism, Maastricht University Medical Centre+ (MUMC+), Maastricht, the Netherlands; 2 Department of Nuclear Medicine, Maastricht University Medical Centre+ (MUMC+), Maastricht, the Netherlands

## Introduction

Brown adipose tissue (BAT) has emerged as a potential target in the treatment and prevention of obesity and type 2 diabetes. We have recently shown that a 10-day cold acclimation period leads to recruitment of BAT in young, lean humans. In addition, rodent studies have shown that prolonged cold exposure and increased BAT volume are associated with improvements in glucose homeostasis and insulin sensitivity. Here, we investigated the effect of cold-acclimation on BAT activity, energy metabolism, and whole-body insulin sensitivity in healthy lean subjects, obese subjects and type 2 diabetic (T2D) patients.

## Methods

Subjects included 17 lean subjects (8 males), 10 obese males and 8 overweight/obese type 2 diabetic male patients (Lean: BMI: 21.6 ± 2.2 kg/m^2^, age 23.0 ± 3.2 y; Obese: BMI: 34.2 ± 4 kg/m^2^, age: 31.8 ± 12.9 y; T2D: BMI: 29.8 ± 23.2 kg/m^2^, age 59.3 ± 5.8 y). BAT activity was assessed by [^18^F]FDG-PET/CT scanning, energy metabolism by indirect calorimetry during nonshivering cold exposure, and insulin sensitivity was measured using a 1-step hyperinsulinemic euglycemic clamp (in T2D patients only) before and after a 10-day cold acclimation period. The cold acclimation period consisted of intermittent cold exposure (air and wall temperature: 14-15 °C, 6 hours/day) for 10 consecutive days. Subjects were wearing shorts, T-shirt, socks and shoes and were engaged in sedentary activities.

## Results

Before acclimation BAT activity was most pronounced in the lean subject group, with lower levels in the obese and type 2 diabetics (BAT activity in SUVmax [maximal standard uptake value] before acclimation: Lean 15.9 ± 5.8, obese 7.4 ± 5.2, T2D 0.40 ± 0.29. In all groups BAT activity was significantly related to nonshivering thermogenesis. The 10-day cold acclimation protocol increased BAT activity significantly (BAT activity in SUVmax after acclimation: Lean 19.9 ± 6.3, obese 15.4 ± 9.3, T2D 0.63 ± 0.78). However, the most pronounced changes were observed in the lean subjects. Interestingly, in the T2D group glucose infusion rate during the hyperinsulinemic euglycemic clamp increased from 15.1 ± 4.9 ml/kg/min before to 21.4 ± 7.6 ml/kg/min after cold acclimation (p < 0.05), indicating pronounced improvements in insulin sensitivity.

## Discussion

Cold acclimation increased brown fat activity and NST in lean subjects with similar, albeit less pronounced, effects in obese and T2D subjects. There was a significant increase in whole-body insulin-mediated glucose disposal in type 2 diabetic patients. Long-term effects and possible involvement of other tissues than BAT in these improvements await further investigation.

## Conclusion

The results indicate that intermittent mild cold exposure could be used as an effective therapy to increase brown fat activity and energy expenditure in lean and obese subjects and improve insulin sensitivity in T2D.

